# Septische Kardiomyopathie – Diagnostik und Schweregradabschätzung

**DOI:** 10.1007/s00063-024-01109-z

**Published:** 2024-02-12

**Authors:** Ursula Müller-Werdan, Alexander Vogt, Karl Werdan

**Affiliations:** 1 Medizinische Klinik für Geriatrie und Altersmedizin, Charité – Universitätsmedizin Berlin und EGZB Berlin, Berlin, Deutschland; 2 Klinik und Poliklinik für Innere Medizin 3, Universitätsklinikum Halle (Saale), Martin-Luther-Universität Halle-Wittenberg, Halle (Saale), Deutschland

**Keywords:** Linksventrikuläre Auswurffraktion (LVEF), „Afterload-related cardiac performance“ (ACP), Sinustachykardiomyopathie, Myokardialer Performanzindex (MPI), Ventrikuloarterielle Kopplung (VAC), Left ventricular ejection fraction (LVEF), Afterload-related cardiac performance, Sinus-tachycardiomyopathy, Myocardial performance index, Ventriculoarterial coupling

## Abstract

**Hintergrund:**

Die septische Kardiomyopathie (sKM) wird in ihrer Relevanz häufig unterschätzt. Die Unterschätzung basiert auf der komplexen Schädigung des Herzens und der Schwierigkeit, den Schweregrad der Funktionseinschränkung zu quantifizieren.

**Ziel der Arbeit:**

Darstellung der methodischen Möglichkeiten zur Diagnosestellung und Schweregradquantifizierung der sKM.

**Methodik:**

Literatursichtung und Analyse der wesentlichen Ergebnisse.

**Ergebnisse:**

Die sKM ist charakterisiert sowohl durch eine systolische als auch diastolische Funktionsstörung nicht nur des linken, sondern auch des rechten Ventrikels sowie durch eine Sinustachykardiomyopathie (≥ 90–95 Schläge/min) variablen Ausmaßes. „Sepsis-related organ failure assessment“ (SOFA)Score, linksventrikuläre Auswurffraktion (LVEF), EKG und kardiale Biomarker sind zur Schweregradquantifizierung nicht hilfreich. Erforderlich dazu ist entweder eine „komplexe“ Echokardiographiediagnostik oder die Bestimmung globaler Herzfunktionsparameter, die die Nachlastabhängigkeit des Herzzeitvolumens (HZV) bei der ausgeprägten Vasodilatation in der Sepsis und im septischen Schock berücksichtigen. Ein entsprechender, mittels HZV-Messung zu ermittelnder Parameter ist „afterload-related cardiac performance“ (ACP), der den Prozentsatz des HZV des Sepsispatienten bei dem jeweiligen Gefäßwiderstand in Relation zum HZV eines gesunden Herzens angibt. Die ACP-Bestimmung zeigt, dass mindestens jeder zweite Sepsispatient eine Herzfunktionseinschränkung aufweist und dass diese mit zunehmendem Schweregrad die Sterblichkeit erhöht.

**Diskussion:**

Einfache Parameter wie die LVEF sind zur Diagnostik und Schweregradklassifizierung der sKM nicht hilfreich. Dazu sind entweder „komplexe“ Echokardiographiemessungen oder – am besten validiert – die ACP-Bestimmung geeignet.

**Zusatzmaterial online:**

Zusätzliche Informationen sind in der Online-Version dieses Artikels (10.1007/s00063-024-01109-z) enthalten.

## Abstract (en)

**Background:**

The relevance of septic cardiomyopathy is frequently underestimated due to the complexity of the pattern of cardiac injury and the corresponding difficulties in quantifying the degree of functional impairment.

**Aim:**

Account of the methods for diagnosis and severity classification of septic cardiomyopathy.

**Methods:**

Literature review and analysis of the main findings.

**Results:**

Septic cardiomyopathy is characterized by both systolic and diastolic impairment of not only the left, but also the right ventricle, as well as by sinus-tachycardiomyopathy (≥ 90–95 beats/min) of variable degree. Sepsis-related organ failure assessment (SOFA) score, left ventricular ejection fraction (LVEF), ECG and cardiac biomarkers do not help in grading severity of septic cardiomyopathy. For that purpose either a sophisticated echocardiography diagnosis is mandatory, or the measurement of those global heart function parameters which take into account the dependency of cardiac output on afterload, in view of the pronounced vasodilatation in sepsis and septic shock, is needed. A suitable parameter on the basis of cardiac output measurement is afterload-related cardiac performance (ACP), which gives the percentage of cardiac output in a septic patient related to the cardiac output a healthy heart pumps when challenged by a fall in systemic vascular resistance to the same extent. The calculation of ACP shows that at least one in two septic patients suffers from impaired heart function and that mortality increases as severity increases.

**Conclusion:**

Simple parameters like LVEF are not apt for diagnosis nor for disease severity classification of septic cardiomyopathy. For that purpose either sophisticated echocardiography techniques or load-independent parameters—best validated—ACP measurements are appropriate.

## Hintergrund

Der septische Schock ist nicht nur Folge eines Kreislaufschocks, sondern auch einer septischen Kardiomyopathie. Zur Quantifizierung dieser Herzschädigung ist die Bestimmung der linksventrikulären Auswurffraktion nicht zielführend; vielmehr muss die Abhängigkeit des Herzzeitvolumens von der septischen Vasoplegie (Nachlastsenkung) berücksichtigt werden. Die Messung der „afterload-related cardiac performance“ (ACP) berücksichtigt diese Abhängigkeit und zeigt an, welchen Prozentsatz das Herz des Septikers im Vergleich zum Herzzeitvolumen eines gesunden Herzens noch leisten kann.

## Septischer Kreislaufschock und septische Kardiomyopathie

Sepsis^1^ und septischer Schock stellen eine der Haupttodesursachen auf Intensivstationen dar mit weiterhin hoher Letalität und ohne nennenswerte Therapiefortschritte in den letzten Jahren. Der septische Schock wird heutzutage immer noch überwiegend als Kreislaufschock gewertet mit Gefäßparalyse und konsekutiver Hypotonie, Gefäß-Leakage und vermindertem Ansprechen auf Vasopressoren. Der Herzschädigung in der Sepsis wird dagegen weit weniger Beachtung geschenkt, obwohl diese zum Teil sehr ausgeprägte Herzfunktionseinschränkung bereits vor mehr als 30 Jahren von Parrillo und Mitarbeitern ausführlich beschrieben worden ist [19]. Diese Herzschädigung in der Sepsis lässt sich als „septische Kardiomyopathie“ klassifizieren, als eine sekundäre Form der Kardiomyopathie mit der Schädigung des Organs Herz im Rahmen der Systemerkrankung Sepsis [15].

## Septische Kardiomyopathie – nicht nur eine systolische Herzinsuffizienz!

**Abb. 1:**
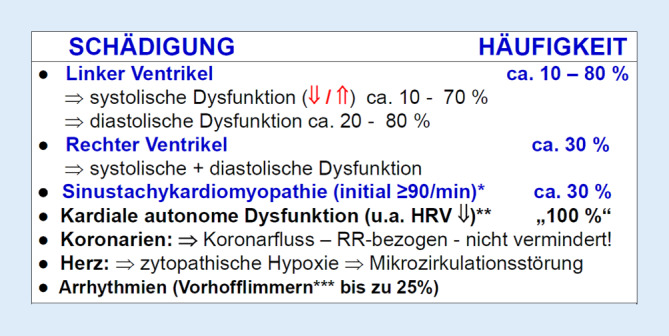
Septische Kardiomyopathie – Formen der Funktionseinschränkung. „HÄUFIGKEIT“: Literaturzusammenstellung; *[10]; **[20]; ***[3]. Ausführliche Informationen in [1, 25, 26]

Das Schädigungsmuster der septischen Kardiomyopathie ist komplex und individuell unterschiedlich schwer ausgeprägt (Abb. 1; [1, 16, 26]). Es findet sich nicht nur eine systolische, sondern auch eine diastolische Funktionseinschränkung sowohl des linken als auch des rechten Ventrikels. Besteht darüber hinaus initial – noch ohne Katecholamintherapie – eine Sinustachykardiomyopathie mit Herzfrequenzen ≥ 90–95 Schlägen/min, so verschlechtert sich die Prognose weiterhin. Die Sinustachykardiomyopathie ist Ausdruck einer kardialen autonomen Dysfunktion, die man an der hochgradig eingeschränkten Herzfrequenzvariabilität erkennen kann. Dabei ist der koronare Blutfluss blutdruckbezogen nicht vermindert, sondern sogar eher aufgrund der dilatierten Koronargefäße gesteigert. Allerdings muss davon ausgegangen werden, dass auch am Herzen Mikrozirkulationsstörungen vorliegen und ebenso eine zytopathische Hypoxie (gestörte Sauerstoffverwertung aufgrund einer Mitochondrienschädigung durch Sepsimediatoren; [16, 25, 26]).

Erfreulicherweise kann die septische Kardiomyopathie innerhalb von wenigen Tagen reversibel sein [16].

## Wenig hilfreich: Leitlinien, SOFA-Score, linksventrikuläre Auswurffraktion (LVEF), ...

Bei einer so komplexen Organschädigung wie der septischen Kardiomyopathie ist nicht zu erwarten, dass mit einem einzelnen Parameter der Schweregrad der Funktionseinschränkung ausreichend abgeschätzt werden kann. Dementsprechend findet sich weder in der internationalen [7] noch in der nationalen [4] *Sepsisleitlinie* eine konkrete Angabe dazu. Und auch der *SOFA-Score* [23] hilft hier nicht weiter: Mit der Punktevergabe der kardiovaskulären Komponente für Blutdruck und Intensität der Vasopressorentherapie lässt sich zwar das Ausmaß des septischen Kreislaufschocks abschätzen, keinesfalls aber der Schweregrad der septischen Kardiomyopathie!

**Abb. 2:**
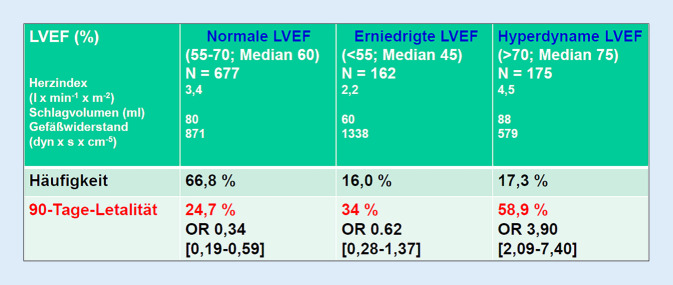
Prognostische Bedeutung der linksventrikulären Auswurffraktion (*LVEF*) bei 1014 Patienten mit Sepsis (21,4 % septischer Schock; 90 Tage-Letalität 32,1 %). Zusammenstellung der Daten nach [5]

Bedauerlicherweise hilft auch die echokardiographische Bestimmung der *linksventrikulären Auswurffraktion (LVEF)* nicht weiter (Abb. 2): Nicht eine erniedrigte LVEF war mit einer signifikant höheren Letalität korreliert, sondern eine hyperdynamische LVEF! Als Erklärung dafür schlagen die Autoren der Studie [5] die unkontrollierte Vasoplegie bei den Patienten mit hyperdynamischer LVEF vor, erkennbar an dem stark erniedrigten Gefäßwiderstand (Abb. 2).

Spezifische *EKG-Zeichen* der septischen Kardiomyopathie gibt es nicht. Im *Röntgenbild des Thorax* kann sich eine transiente Herzvergrößerung [16] zeigen. Bei 30–85 % der Sepsispatienten finden sich zumindest leicht erhöhte *Troponinwerte* mit höheren Werten (Troponin I 0–160 ng/ml) bei höhergradiger Herzschädigung als bei fehlender Herzschädigung (Troponin I 0–40 ng/ml), allerdings bei breiter Überlappung [24, 28]. *Natriuretische Peptide* können in der Sepsis beträchtliche Anstiege zeigen (Median 6000 pg/ml; Interquartilenbereich 1000–16.000 pg/ml); bei systolischer/diastolischer Dysfunktion finden sich höhere Werte (Median 10.000 pg/ml) als bei fehlender kardialer Dysfunktion (Median 1000 pg/ml; [12]).

## Alternativen – „komplexe“ Echokardiographie und (weitgehend) lastunabhängige globale Parameter

### „Komplexe“ Echokardiographie

**Abb. 3:**
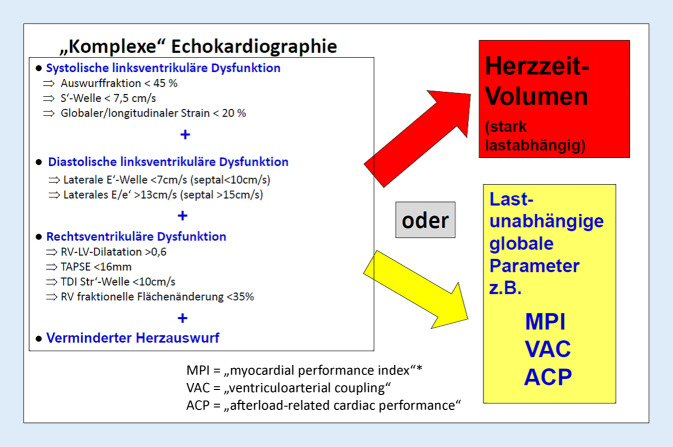
Erfassung der Pumpfunktionseinschränkung der septischen Kardiomyopathie mittels „komplexer“ Echokardiographie und mittels globaler Pumpfunktionsparameter. Die Zusammenstellung der echokardiographischen Parameter im Sinne der „komplexen“ Echokardiographie erfolgte nach [2]. *Vorlastunabhängig, aber nachlastabhängig

Will man die Pumpfunktionseinschränkung der septischen Kardiomyopathie echokardiographisch möglichst in Gänze erfassen, so müssen systolische und diastolische Funktion sowohl des linken als auch des rechten Ventrikels erfasst werden, was nur unter Einbeziehung von Parametern der Gewebe-Doppler-Echokardiographie als auch des „strain/speckle tracking“ möglich ist. Ein entsprechender Vorschlag von Boissier und Aissaoui [2] findet sich in Abb. 3, wobei diese Autoren aber auch auf die Limitationen dieses Vorgehens vor allem bei Intensivpatienten und die erforderliche echokardiographische Erfahrung der Untersucher nachdrücklich hinweisen. Und selbst bei optimaler Erfassung aller Parameter stellt sich die bisher ungelöste Frage, in welcher Weise die Ergebnisse in eine Aussage zum Schweregrad der Herzfunktionseinschränkung gebracht werden können. Somit ist es mit der „komplexen“ Echokardiographie auch nicht möglich, eine Aussage zur prognostischen Bedeutung der Herzschädigung zu treffen.

### (Weitgehend) lastunabhängige globale Parameter

**Abb. 4:**
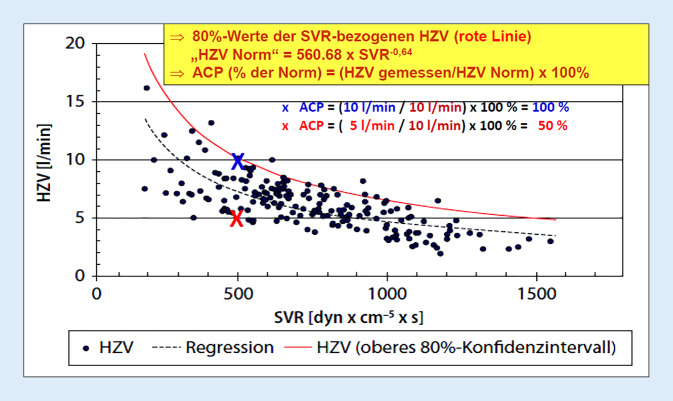
Berechnung der nachlastbezogenen Herzperformanz („afterload-related cardiac performance“, *ACP*) bei 24 Patienten mit septischem Multiorgandysfunktionssyndrom (*MODS*). Bei 24 Patienten mit septischem MODS wurde mehrfach im Krankheitsverlauf das HZV gemessen [24]. Die *durchgezogene rote Linie* ({HZV Norm} = 560,68 × {SVR}^−0,64^) entspricht dem HZV (oberes 80 %-Konfidenzintervall) eines nicht oder nicht wesentlich durch die Sepsis geschädigten Herzens des Sepsispatienten bei dem jeweiligen SVR-Wert. Aufgetragen wurden die gemessenen HZV-Werte gegen die jeweiligen berechneten SVR-Werte. Weitere Erläuterungen siehe Text und [24]. *ACP* „afterload-related cardiac performance“, *HZV* Herzzeitvolumen, *SVR* „systemic vascular resistance“ (Ergänzt nach [24])

In der Sepsis und vor allem im septischen Schock kann das Herzzeitvolumen (HZV) nicht isoliert betrachtet werden, sondern nur – aufgrund der sepsisbedingten Vasoplegie mit Vasodilatation – in Relation zum systemischen Gefäßwiderstand („systemic vascular resistance“, SVR): Mit zunehmender Absenkung des SVR muss zur Stabilisierung des Blutdrucks das HZV kompensatorisch ansteigen (Abb. 4). Demzufolge muss das HZV um die Lastabhängigkeit korrigiert werden. Dies ist mit den Parametern „myokardialer Performanzindex“ („myocardial performance index“, MPI), „ventrikuloarterielle Kopplung“ („ventriculoarterial coupling“, VAC) und nachlastbezogene Herzperformanz („afterload-related cardiac performance“, ACP) mehr oder weniger möglich [1, 2].

⇒ *MPI*: LV-MPI und RV-MPI sind definiert als die mittels Gewebe-Doppler bestimmte Summe der isovolumetrischen Kontraktions- und Relaxationszeit dividiert durch die Auswurfzeit. Geringere Werte (LV-MPI normalerweise etwa 0,5) zeigen eine bessere globale LV-Funktion an. LV-MPI ist unabhängig von der Vorlast, der Herzfrequenz und moderatem PEEP [18], aber abhängig von der Nachlast [13]. In Beobachtungsstudien wurde sowohl dem LV-MPI [18] als auch dem RV-MPI [9] eine prognostische Bedeutung zugeschrieben.

⇒ *VAC *beschreibt das Verhältnis von arterieller (Ea) und ventrikulärer (Ees) Elastanz. Die Bestimmung der VAC (Ea/Ees) ist sowohl invasiv als auch echokardiographisch möglich [17]. Der Normalwert liegt bei 1 ± 0,36 (Median ± Interquartilenbereich; [8]). Zumindest jeder vierte Patient mit Sepsis/septischem Schock zeigt initial einen erhöhten Ea/Ees-Wert ≥ 1,36 [8, 17]. Eine Abnahme des erhöhten Ea/Ees-Werts korrelierte bei Patienten mit septischem Schock mit einer Zunahme des Schlagvolumens und einer Abnahme der erforderlichen Noradrenalindosierung [14].

⇒ Der Stellenwert der *ACP-Bestimmung* wird im nächsten Abschnitt ausführlich beschrieben.

## Septische Kardiomyopathie – ACP quantifiziert Schweregrad

Sepsis und septischer Schock sind durch eine ausgeprägte Vasoplegie mit Vasodilatation und einem erniedrigten SVR (Normalwert ca. 1000–1100 dyn × cm^−5^ × s) mit Blutdruckabfall charakterisiert. Zur Blutdruckstabilisierung kann ein nicht durch die Sepsis geschädigtes Herz bei SVR-Abfall sein HZV beträchtlich steigern (rote Linie in Abb. 4). Für einen bestimmten SVR-Wert findet sich allerdings bei Sepsispatienten eine breite Streuung der HZV-Werte als Ausdruck einer unterschiedlich ausgeprägten Herzfunktionseinschränkung. In Relation zur roten Linie kann für jeden gemessenen HZV-Wert berechnet werden, welchen Anteil in Prozent das sepsisgeschädigte Herz in Relation zum HZV eines gesunden Herzens noch erbringen kann. Dieser prozentuale Anteil wird durch „afterload-related cardiac performance“ (ACP) wiedergegeben. Für einen SVR von 500 dyn × cm^−5^ × s liegt das HZV eines gesunden Herzens bei 10 l × min^−1^. Liegt das HZV des Sepsispatienten bei diesem SVR bei 5 l × min^−1^, so beträgt der ACP-Wert 5 l × min^−1^/10 l × min^−1^ × 100 % = 50 %. Beträgt das gemessene HZV des Sepsispatienten bei diesem SVR dagegen 10 l × min^−1^, so beträgt der ACP-Wert 10 l × min^−1^/10 l × min^−1^ × 100 % = 100 %, sodass bei diesem Patienten keine sepsisbedingte Schädigung der Herzfunktion vorliegen würde (ACP = 100 %).

Auf der Intensivstation wird das HZV üblicherweise invasiv (PiCCO, Pulmonalarterienkatheter) gemessen. Aber auch die nichtinvasive Bestimmung z. B. in der Notaufnahme mittels Task Force® Monitor (CNsystems, Graz, Österreich) ist möglich [27]. Die ACP-Berechnung (Abb. 4; siehe auch Zusatzmaterial online) kann in Patientendatenmanagementsysteme integriert werden, sodass bei Eingabe von Blutdruck (RR), HZV und zentralem Venendruck (ZVD) Werte für HZV, SVR und ACP angezeigt werden.

Neben der Nachlastabhängigkeit des HZV bei Septispatienten beeinflussen sicherlich auch noch weitere Faktoren – vor allem Vorlast, Herzfrequenz, wahrscheinlich auch Alter und Geschlecht – in nicht unerheblichem Maße die Herzleistung des Sepsispatienten. Unser aktuelles ACP-Konzept berücksichtigt derzeit explizit nur die Nachlast. Der Einfluss der Vorlast wird bei einer adäquaten Volumensubstitution abgeschwächt und darüber hinaus geht der zentrale Venendruck in die Berechnung des SVR mit ein (siehe Zusatzmaterial online).

Nach der Validierung und Erstbeschreibung der ACP-Bestimmung [24] haben mittlerweile mehrere Arbeitsgruppen das ACP-Konzept aufgegriffen und dessen prognostische Relevanz bei Sepsis/septischem Schock bestätigt (siehe nächsten Abschnitt; [6, 22, 27, 30]). Und auch bei der chronischen Herzinsuffizienz liefert die ACP-Bestimmung valide Informationen zur Prognose der Patienten [29].

## Die septische Kardiomyopathie erhöht das Letalitätsrisiko!

Anhand des ACP-Werts wurde das Ausmaß der Funktionseinschränkung der septischen Kardiomyopathie in der Erstbeschreibung [24] folgendermaßen klassifiziert: ACP > 80 %: keine („no“) Funktionseinschränkung; 60 % < ACP ≤ 80 %: leichte („slight“) Funktionseinschränkung; 40 % < ACP ≤ 60 %: mäßige („moderate“) Funktionseinschränkung; ACP ≤ 40 %: schwere („severe“) Funktionseinschränkung.

**Abb. 5:**
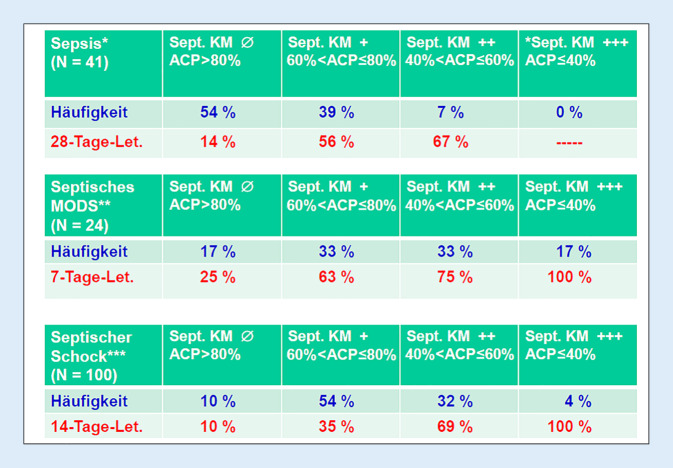
Häufigkeit und Letalität der septischen Kardiomyopathie. *Zusammenstellung nach [30]. **Zusammenstellung nach [24]. ***Zusammenstellung nach [6]. ∅, +, ++, +++ charakterisieren den Schweregrad der septischen Kardiomyopathie als Funktionseinschränkung nach [24]: ∅: keine („no“) Funktionseinschränkung; +: leichte („slight“) Funktionseinschränkung; ++: mäßige („moderate“) Funktionseinschränkung; +++: schwere („severe“) Funktionseinschränkung. *ACP* „afterload-related cardiac performance“, *KM* Kardiomyopathie, *MODS* Multiples Organdysfunktionssyndrom, *Sept.* septische

Abb. 5 zeigt in 3 Studien Häufigkeit und 28-Tage-Letalität der verschiedenen Funktionseinschränkungen der septischen Kardiomyopathien klassifiziert anhand des ACP-Werts: Bei allen 3 Studien ist die 28-Tage-Sterblichkeit bei Fehlen einer septischen Kardiomyopathie (ACP > 80 %) am geringsten und nimmt mit zunehmender Herzfunktionseinschränkung – abnehmendem ACP-Wert – deutlich zu. Bei Sepsispatienten hat nur jeder zweite Patient keine Herzfunktionseinschränkung (ACP > 80 %). Bei Patienten mit septischem Multiorgandysfunktionssyndrom (MODS; Abb. 5: septisches MODS) und bei Patienten mit septischem Schock (Abb. 5: septischer Schock) liegt der Anteil der Patienten mit fehlender Herzfunktionseinschränkung (ACP > 80 %) weit unter 50 %.

Aktuell besitzt die ACP-Bestimmung bei septischen Patienten die höchste Aussagekraft in Bezug auf die prognostische Bedeutung der septischen Kardiomyopathie, sie ist anderen globalen Parametern wie Herzzeitvolumen oder „cardiac power output“ überlegen [27, 29, 30].

Bei Patienten mit Sepsis noch ohne Organdysfunktion (nach alter Nomenklatur „Sepsis“ im Gegensatz zu „schwere Sepsis“ und „septischer Schock“) – z. B. in der Notaufnahme – haben mehr als drei Viertel der Patienten eine regelrechte Herzfunktion (ACP > 80 %) und die Sterblichkeit selbst der Patienten mit leichter Herzfunktionseinschränkung (60% < ACP ≤ 80%) ist mit etwa 10 % nur gering [25, 27].

## Diagnostik der septischen Kardiomyopathie – Wie vorgehen?

In den Arztbriefen von Patienten mit Sepsis und septischem Schock findet sich nur selten die Organdysfunktion „septische Kardiomyopathie“, wohingegen „ARDS“ und „akutes Nierenversagen“ häufig aufgeführt sind. Dies liegt nicht daran, dass die septische Kardiomyopathie seltener auftritt als das ARDS und das akute Nierenversagen, sondern daran, dass die Diagnose „septische Kardiomyopathie“ viel zu selten gestellt wird! Grund dafür ist vor allem, dass diese Diagnose aufgrund der Komplexität dieser Organdysfunktion (Abb. 1) mit einfachen echokardiographischen Parametern wie der linksventrikulären Auswurffraktion nicht abgebildet werden kann (Abb. 2). Erforderlich sind vielmehr globale Herzfunktionsparameter, die die Auswirkungen der massiven Nachlastsenkung im septischen Schock auf das Herzzeitvolumen berücksichtigen (Abb. 3). Hierbei zeigt aktuell die ACP-Bestimmung (Abb. 4) die größte Trennschärfe in Bezug auf Schweregrad und prognostische Relevanz der septischen Kardiomyopathie: ACP gibt das Herzzeitvolumen des Patienten als Prozentsatz des Herzzeitvolumens eines gesunden Herzens wieder unter Berücksichtigung der HZV-Abhängigkeit vom systemischen Gefäßwiderstand (SVR). Damit wird ersichtlich, in welchem globalen Ausmaß das HZV des Sepsispatienten aufgrund der systolischen und diastolischen Dysfunktion von linkem und rechtem Ventrikel sowie einer bestehenden Tachykardiomyopathie (Abb. 1) tatsächlich eingeschränkt ist. Die ACP-Bestimmung verdeutlicht auch in eindrucksvoller Weise, in welchem Ausmaß ein höherer Schweregrad der septischen Kardiomyopathie zur Sterblichkeit der Sepsispatienten beiträgt (Abb. 5).

Der potenzielle Nutzen der ACP-Bestimmung rechtfertigt bei Sepsispatienten mit hoher Sterblichkeit auch den Einsatz einer invasiven HZV-Messung [11] mittels Pulmonalarterienkatheter oder PiCCO zur HZV-Messung und ACP-Bestimmung, wobei zur ACP-Bestimmung bereits auch weniger invasive (FloTrac/Vigileo^TM^, Edwards Lifesciences, Irvine, CA, USA) sowie nichtinvasive HZV-Techniken (Task Force® Monitor System, CNsystems, Graz, Österreich) zum Einsatz gekommen sind [27].

Die Quantifizierung des Schweregrads der septischen Kardiomyopathie mittels ACP ist kein Selbstzweck: Im Krankheitsverlauf zeigt die Änderung des ACP-Werts das Ansprechen auf eine eingeleitete Therapie und eine Erholung der Herzfunktion an.

Dass bislang keine relevante medikamentöse Therapie der septischen Kardiomyopathie in Sicht ist, liegt auch daran, dass der Erfolg einer Therapie auf die Besserung der Herzfunktion bisher nicht überzeugend dokumentiert werden konnte, was nun aber mit der ACP-Bestimmung möglich ist. Und auch in bereits publizierten Therapiestudien ließen sich bei Patienten mit vorhandener HZV-Messung mittels nachträglicher ACP-Bestimmung Informationen zur Wirksamkeit der Behandlung auf die Herzfunktion ermitteln.

## Fazit für die Praxis

- Der septische Schock wird häufig als Kreislaufschock und weniger als Schädigung des Herzens angesehen.
- Die septische Kardiomyopathie – obwohl mindestens bei jedem zweiten Sepsispatienten relevant – ist unterdiagnostiziert, da der Echokardiographieparameter „linksventrikuläre Auswurffraktion“ diesbezüglich nicht weiterhilft.
- Dagegen erlaubt die Bestimmung der „afterload-related cardiac performance“ (ACP) via Herzzeitvolumen eine Quantifizierung des Schweregrads der septischen Kardiomyopathie und damit auch eine Abschätzung des Letalitätsrisikos infolge des Ausmaßes der septischen Kardiomyopathie. Der ACP-Wert kann invasiv, aber auch nichtinvasiv bestimmt und in Patientendatenmanagementsysteme installiert werden.
- Die ACP-Bestimmung erlaubt somit den Nachweis des Vorliegens einer septischen Kardiomyopathie, die Quantifizierung deren Schweregrads im Krankheitsverlauf und die Beurteilung des Ansprechens auf eine Therapie.

## Supplementary Information

Berechnung von SVR und ACP anhand der Formel der Originalpulikation [24] (linke Spalte): Eingabe von Herzzeitvolumen in l/min (CO), mittlerem arteriellen Blutdruck in mm Hg (MAP) und zentralem Venendruck in mm Hg (CVP): Das Programm berechnet daraus den systemischen Gefäßwiderstand (SVR) und die Afterload-related Cardiac Performance (ACP). Alternativ kann mit CO und SVR der ACP-Wert berechnet werden (rechte Spalte).

## Footnotes

1 Gemeint mit „Sepsis“ ist durchgehend im Text die aktuelle Sepsisdefinition „lebensbedrohliche Organdysfunktion verursacht durch eine fehlgeleitete Wirtsaktion auf die Infektion“ (Sepsis‑3, [21]), die in etwa der früheren Definition der „schweren Sepsis“ entspricht.
